# Mixed-Effects Model to Assess the Effect of Disengagements on Speed of an Automated Shuttle with Sensors for Localization, Navigation, and Obstacle Detection

**DOI:** 10.3390/s25020573

**Published:** 2025-01-20

**Authors:** Abhinav Grandhi, Ninad Gore, Srinivas S. Pulugurtha

**Affiliations:** 1 Department of Civil & Environmental Engineering, The University of North Carolina at Charlotte, Charlotte, NC 28223, USA; agrandhi@charlotte.edu (A.G.); ninadgore24@gmail.com (N.G.); 2 Department of Civil Engineering, The University of Mississippi, University, MS 38677, USA

**Keywords:** automated shuttle, disengagement, speed, sensors

## Abstract

The focus of this study is to investigate the underexplored operational effects of disengagements on the speed of an automated shuttle, providing novel insights into their disruptive impact on performance metrics. For this purpose, global positioning system data, disengagement records, weather reports, and roadway geometry data from an automated shuttle pilot program, from July to December 2023, at the University of North Carolina in Charlotte, were collected. The automated shuttle uses sensors for localization, navigation, and obstacle detection. A multi-level mixed-effects Gaussian regression model with a log-link function was employed to analyze the effect of disengagement events on the automated shuttle speed, while accounting for control variables such as roadway geometry, weather conditions, time-of-the-day, day-of-the-week, and number of intermediate stops. When these variables are controlled, disengagements significantly reduce the automated shuttle speed, with the expected log of speed decreasing by 0.803 units during such events. This reduction underscores the disruptive impact of disengagements on the automated shuttle’s performance. The analysis revealed substantial variability in the effect of disengagements across different route segments, suggesting that certain segments, likely due to varying traffic conditions, road geometries, and traffic control characteristics, pose greater challenges for autonomous navigation. By employing a multi-level mixed-effects model, this study provides a robust framework for quantifying the operational impact of disengagements. The findings serve as vital insights for advancing the reliability and safety of autonomous systems through targeted improvements in technology and infrastructure.

## 1. Introduction

Automated shuttles are significant additions to the future of autonomous public transportation systems, offering the potential to revolutionize urban mobility. These vehicles are particularly valuable in urban environments and campus settings, where they can serve as seamless links between major transit hubs and final destinations. Their ability to operate in dedicated lanes or mixed traffic conditions further underscores their versatility and transformative potential in providing public transportation.

Over the past decade, more than 120 pilot deployments of automated shuttles have been undertaken globally, demonstrating their utility in diverse settings such as university campuses, airports, recreational parks, business parks, and high-pedestrian areas [1,2]. These deployments were aimed at various purposes, including first-mile-last-mile connectivity, mobility enhancement, and accessibility improvements. Automated shuttles offer numerous benefits when integrated into public transportation systems, including reduced congestion, enhanced accessibility, and lower environmental impact. Additionally, they hold the promise of improving mobility and access for regular transportation system users as well as transportation-disadvantaged groups, such as the elderly, people with disabilities, and non-driving age groups, thereby bridging social inequities [3,4].

Automated shuttles embody Level 3 or Level 4 autonomous technology, characterized by the absence of traditional driving controls such as steering wheels and pedals. This indicates complete reliance on autonomous systems for all driving-related tasks and decision-making. This high level of automation enables shuttles to perform complex driving functions autonomously, such as navigation, obstacle detection, and collision avoidance. However, despite these capabilities, there are situations where the automated system may not be able to handle specific driving scenarios, leading to disengagements.

Disengagements involve a transfer of control from the automated system to a human operator, typically due to interruptions in the shuttle’s decision-making process. Several factors can cause these interruptions. Automated shuttles rely heavily on continuous communication with Global Positioning Systems (GPS) satellites and other signal sources to navigate accurately; signal loss or degradation can result in the autonomous system being unable to determine its precise location, necessitating human intervention. Additionally, vehicle-to-infrastructure (V2I) communication failures can disrupt the shuttle’s ability to make informed driving decisions, as these shuttles use V2I communication to receive real-time information from road infrastructure, such as traffic signals and road signs. The behavior of other road users, such as pedestrians, cyclists, and other vehicles, can be unpredictable. Sudden or erratic movements by these road users can present situations that the autonomous system is not equipped to handle, requiring a safety operator to take control. Moreover, adverse weather conditions such as heavy rain, snow, fog, or ice can impair the sensors and cameras used by automated shuttles for navigation and obstacle detection. Poor visibility and slippery roads can reduce the effectiveness of the autonomous system, leading to disengagements [5]. These transfers of control, initiated by either the shuttle (passive) or the operator (active), can significantly impact the shuttle’s operational performance, particularly its speed.

Most related studies in the past focused on the disengagement of automated vehicles from a safety perspective, examining the conditions under which they occur and how they influence vehicle and road-user safety [6,7,8,9,10,11]. However, there is a gap in understanding the effect of disengagements on operational efficiency. This gap is critical because frequent disengagements can cause interruptions in service, reduce the shuttle’s average speed, and affect overall travel time. Moreover, each disengagement event not only introduces variability in the shuttle’s performance, which can have an impact on the scheduling and reliability of shuttle services, but also poses challenges to cost-effectiveness, user satisfaction, and public trust in autonomous technology. Addressing this gap is essential for improving the operational performance and reliability of automated shuttles. This study makes a novel contribution by addressing the underexplored operational effects of disengagements in automated shuttles. By employing a mixed-effects model, this research not only quantifies the effects of disengagements on the automated shuttle’s running speed, but also identifies how roadway and environmental factors have an influence on running speed. The findings provide actionable insights to improve operational efficiency and reliability, with practical implications for the broader adoption of automated shuttle systems in public transportation. With this motivation, the present study addresses the following research questions.

1. How do various factors, such as roadway geometry and operational settings, influence the frequency of disengagements?
2. How do disengagement events affect the running speed of the automated shuttle?

The subsequent sections of this research paper are organized to systematically address the study’s objectives and findings. Section 2 presents a review of the literature, synthesizing existing studies on disengagements while identifying knowledge gaps. Section 3 provides an overview of the UNC Charlotte Autonomous Shuttle Program, detailing its route characteristics and challenges. Section 4 focuses on data collection, outlining the data used for the analysis. Section 5 explains data processing and integration, describing the techniques employed to clean, integrate, and prepare the data for analysis. Section 6 presents the methodology, detailing the application of a mixed-effects model to analyze the impact of disengagements on the automated shuttle’s performance, accounting for variability across operational conditions. Section 8 concludes the study by summarizing key findings, emphasizing the effects of disengagements and roadway factors on the automated shuttle’s performance, discussing limitations, and providing suggestions for future research.

## 2. Literature Review

The rapid development and testing of autonomous vehicles (AVs) on public roads have led to significant insights into the cause and implications of disengagements, situations where vehicle control must be transferred from the automated system to a human driver. An analysis of disengagement data provides crucial information about the performance and safety of AV technologies.

Favaro et al. [12] comprehensively analyzed disengagement data and observed trends in disengagement reporting, including frequencies and average mileage before disengagements occur. They emphasized the importance of understanding disengagements as a safety measure, noting that while they are rare, they serve as critical indicators for improving AV systems and regulations [12]. Lv et al. [5] reviewed disengagement files from major AV manufacturers and classified disengagement events into types, and identified software issues as the most common cause. The research highlighted the need for enhanced takeover mechanisms and time management to improve safety and efficiency [5]. Boggs et al. [13] examined data from California’s AV testing program, focusing on 159,840 disengagements and 124 crashes. They found that AV technology’s maturity and specific triggers, such as hardware and software issues, influenced the likelihood of disengagements [13]. They also emphasized the importance of distinguishing between disengagements initiated by AV systems and those by human operators [13].

Favaro et al. [14] reported that higher speeds at the time of disengagement lead to worse takeover performance, including increased vehicle drift. Dixit et al. [15] analyzed disengagement data to assess the trust and reaction times of human drivers. They found that reaction times to disengagements averaged 0.83 s and varied based on disengagement types and roadway conditions [15]. The increased vehicle miles traveled with improved trust and reaction times is crucial for understanding how driver behavior impacts safety during transitions between automated and manual control [15].

Sinha et al. [16] assessed AV disengagement and crash data from 2014 to 2019 and concluded that a decrease in the number of disengagements does not necessarily indicate improved AV technology. They highlighted issues with current data reporting protocols and recommended improvements to enhance the transparency and effectiveness of AV testing [16]. Khattak et al. [17] analyzed AV disengagement and crash data using statistical models. They identified software failures and planning issues as significant causes of disengagement [17]. They also showed a decrease in the number of disengagements as technology matured [17]. The findings suggest that disengagement is part of AVs’ safety performance and should be monitored closely to avoid potential failures [17]. Wang and Li [18] identified planning issues and sensor limitations as major causes of disengagements. They also highlighted how roadway characteristics and perception issues impact drivers’ response times during disengagements [18].

Zhang et al. [6] analyzed disengagement data using taxonomy, visualization, and statistical tests. They found that (a) manufacturers tested AVs intensively during the Spring and Winter months, (b) test drivers initiated more than 80% of disengagements, while more than 75% of disengagements were because of errors in perception, localization, mapping, planning, and control of the AV system, and (c) there was a significant relationship between the initiator of AV disengagement and the cause category [6]. Houseal et al. [19] used multiple statistical approaches and observed that the latent variables that could identify an AV crash are operator involvement, incorrect maneuver decision, crash severity, and environmental conditions. Based on the AV crash data, Ashraf et al. [7] reported that driving mode (automated vs. non-automated driving conditions) significantly impacts the safety performance of AVs.

Li et al. [8] analyzed the effect of age and disengagement on the takeover control performance of drivers in highly automated vehicles. They revealed that 20 s was sufficient time for drivers to take over control of the AVs after the disengagement [8]. They also revealed that older drivers took longer to respond and make decisions than younger drivers [8]. Wu et al. [9] validated the California DMV AV tester disengagement data and found that disengagements are associated with crash risks. Guo and Zhang [10] reviewed and revealed that by participating in the AV tester program, AV manufacturers showed a trend toward improvement in their AV technologies (e.g., an 8% decrease in the number of disengagements caused by hardware and software discrepancies and a 12% decrease in the number of disengagements caused by perception discrepancies). Gershon et al. [11] concluded that automation-initiated disengagements triggered substantial changes in driver glance behavior, including shorter on-road glances and frequent transitions between road and instrument cluster glance locations. Shirani et al. [20] investigated the driver’s reaction to the disengagement of an advanced driver assistance system. They revealed that disengagements and distraction significantly influence the mean response time of the driver [20].

Overall, past studies have contributed to an understanding of the factors influencing AV disengagements and their implications for safety, driver behavior, and technology development. A notable gap exists in integrating the disengagement data with GPS information to assess the effect of disengagements on the speed of AVs. The disengagement data provide insights into when and why control is transferred from the automated system to a human driver. However, they do not fully capture the vehicle’s performance dynamics across different operational contexts. By integrating the GPS data with the disengagement records, this study aims to bridge the gap, offering a more nuanced evaluation of automated shuttle performance.

## 3. UNC Charlotte Autonomous Shuttle Program

**Figure 1:**
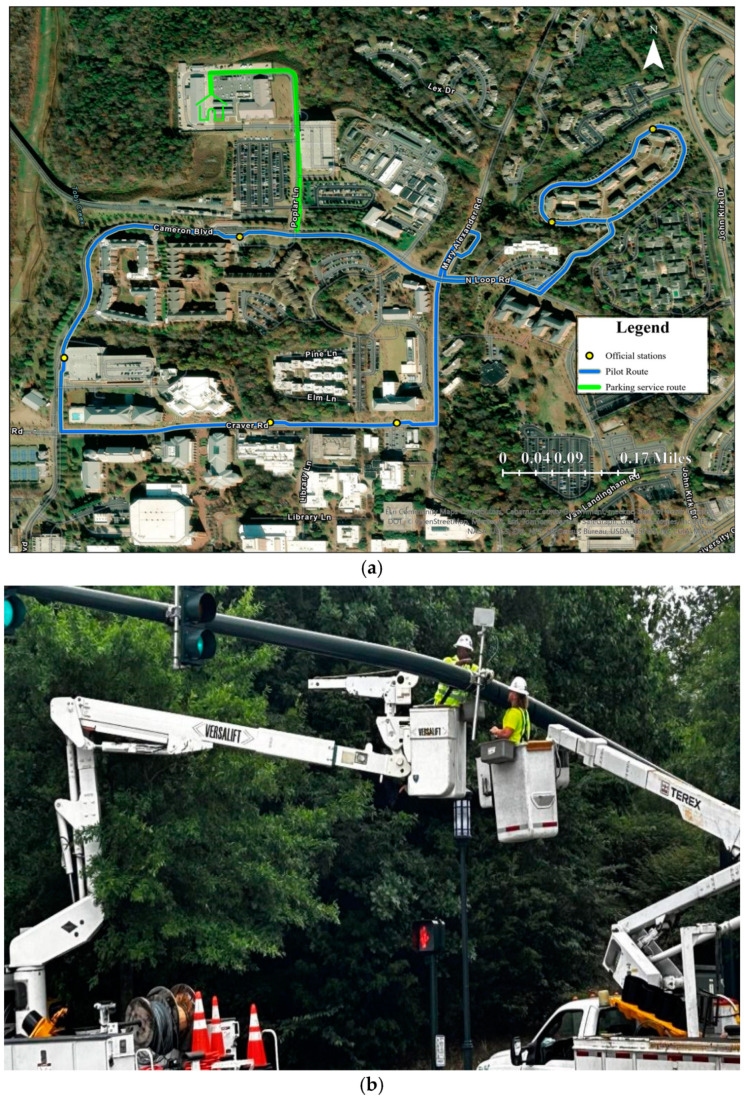
UNC Charlotte automated shuttle program details. (**a**) Pilot route. (**b**) Setting up RSU at a signalized intersection near the LRT station.

The North Carolina Department of Transportation (NCDOT) partnered with UNC Charlotte in 2023 to bring a novel-design, low-speed automated shuttle to campus through the Connected Autonomous Shuttle Supporting Innovation (CASSI) program. As part of the pilot, the automated shuttle operated from July to December 2023 on a dedicated 2.2-mile (3.54 km) route servicing student housing and academic buildings at UNC Charlotte, as shown in Figure 1a. The automated shuttle uses eight light-detection and ranging (LiDAR) sensors, three cameras, one GPS sensor/global navigation satellite system (GNSS) antenna, and one inertial measurement unit (IMU) for localization, navigation, and obstacle detection [21]. Furthermore, Kapsch RIS-9160 [22] with Mobile Mark 6 dB 5.9 Ghz antennas were installed as roadside units to wirelessly communicate with the four traffic signal controllers (2070 controllers retrofitted in June 2023 with 2070-1C CPU modules) along the pilot route. The shuttle made stops at seven key locations: Greek Village 1 (GV1), Greek Village 4 (GV4), Greek Village 8 (GV8), Science Building (SB), Student Union (SU), Student Union Deck (SD), and the Light Rail Transit (LRT) main station.

The selected 2.2-mile (3.54 km) route for the pilot is the most complex route on the campus. It navigates through heterogeneous traffic conditions, including private vehicles, buses, pedestrians, bicyclists, skateboard users, e-scooters, and carts. This route included various traffic control features, such as three signalized intersections, one signalized pedestrian crosswalk, two stop-controlled intersections, and nine unsignalized pedestrian crosswalks. This diverse and dynamic setting offered a robust testbed for evaluating the operational performance of the automated shuttle in real-world conditions. During the pilot period, the automated shuttle operated under various weather conditions, encompassing the hot summer, cool fall, and early winter months, with dry weather and rain. Visibility conditions varied from clear to cloudy and foggy days, providing a comprehensive range of environmental scenarios. However, the automated shuttle was not operational under inclement weather conditions, such as heavy rains or poor visibility.

The automated shuttle was programmed to execute only right turns at intersections, to adhere to National Traffic Safety Administration (NHTSA) guidelines for AV operation. This restriction necessitated a route optimization strategy for the GV4 to SB segment. A signalized intersection near the Student Health Center presented a challenge, due to the left turn required to reach SB directly. To address this, the shuttle turned right into Parking Lot 12, then safely merged onto the main roadway via another right turn, before proceeding toward SB.

To ensure efficient communication with campus traffic signals, roadside units (RSUs) were installed, facilitating the interaction between the automated shuttle and the traffic signal infrastructure. Figure 1b depicts the installation of RSUs at traffic signal controls near the LRT station.

## 4. Data Collection

Data from multiple sensors and sources were collected and integrated for a comprehensive operational performance evaluation of the automated shuttle.

### 4.1. GPS Data

A tablet equipped with the Passio GO App [23] was installed in the automated shuttle to facilitate GPS data collection. The GPS data for the automated shuttle were collected from 13 July 2023 to 15 December 2023. The GPS data were downloaded from the Passio GO database.

The GPS data were obtained as stop-based data, i.e., data were only logged when the automated shuttle stopped, rather than providing continuous data. The recorded data included various parameters such as the start date and time at each stop, the type of activity (always marked as ‘STOP’), the name of the location where the stop occurred, the route being followed, the duration between the previous stop and the current one, the idle time spent at each location, and trip miles. Additionally, the GPS data included the cumulative elapsed time from the start of the pilot program. This comprehensive dataset provided a detailed record of the automated shuttle operations throughout the pilot period. In addition to GPS data, the distance between each official stop was measured to identify intermediate and official stops.

### 4.2. Disengagement Data

The disengagement data for the automated shuttle from July 13 to December 21 were also collected. The operator [24] recorded this data whenever the automated shuttle switched control from an automated mode to a manual mode. These data included detailed incident information such as the incident date and time, the week of the year, the number of weeks into the pilot program, the name of the site where the shuttle was operating, the shuttle ID as per NCDOT records, the route, latitude, and longitude where the incident occurred, weather condition, vehicle speed at the time of the incident, and the cause of the disengagement. The causes of disengagements were fault code/error codes, signal loss, signalized intersection issues, interactions with other road users, station blockages, obstacle detection, vegetation, objects in the priority zone, and manual deviations from the approved path. These disengagement data were used to evaluate the effect of disengagements on the operational performance of the automated shuttle.

### 4.3. Weather Data

Weather data were collected from Visual Crossing [25], an open-source weather data provider, to assess the effect of weather on the operational performance of the automated shuttle. This data were acquired using Visual Crossing’s inbuilt application programming interface (API), ensuring precise and comprehensive coverage throughout the six-month pilot period. The collected data encompassed critical weather parameters, including precipitation, visibility, and overall weather conditions. The precipitation data detailed the intensity and duration of rainfall. Additionally, the weather conditions data provided information on temperature, humidity, and wind speed, which is vital to understand these factors’ potential effects on the automated shuttle’s performance.

### 4.4. Roadway Characteristics Data

Roadway characteristics data along the pilot route were extracted from Google Earth. This included recording each official stop’s latitude and longitude along the route, the distance between each official stop to identify intermediate and official stops, the number of crosswalks, intersections, and access points, and the proportion of divided and undivided segments.

## 5. Data Processing and Integration

### 5.1. Trajectory Construction Framework

The downloaded GPS data from the Passio GO database was processed to construct trajectories of the automated shuttle. These trajectories capture the vehicle’s movement over time and are used to compute performance metrics such as speed, travel time between stops, stop time at each stop, and the number of intermediate stops. The framework for constructing trajectories from the stop-based GPS data is explained next.

### 5.2. Estimation of Key Metrics

- Exit Time: The start time (T_start) for each stop was recorded when the shuttle entered a stop and was provided in the GPS data. Idle time (T_idle) at each stop, available in the GPS data, represents the dwell time or stop time at each stop, indicating how long the automated shuttle remained stationary at each location. The exit time (T_exit) for each stop was calculated by adding the idle time to the start time at each stop. Specifically, the exit time is determined using the formula: T_exit = T_start + T_idle;
- Travel Time Between Stops: The travel time between stops was estimated by subtracting the exit time of the previous stop (i) from the start time of the next stop (i + 1). This is represented by the formula: T_traveltime = Tstart_i + 1 − Texit_i;
- Distance Between Stops: The distance between stops was estimated by subtracting the trip miles (TM) at the previous stop (i) from the TM at the current stop (i + 1). This can be expressed as: D = TM_i + 1 − TM_i.

These metrics were crucial for accurately mapping the automated shuttle’s trajectory and assessing its operational performance.

### 5.3. Identification of Intermediate and Official Stops

Since the automated shuttle made intermediate stops between two official stops, it was imperative to identify whether a stop was an intermediate stop or an official stop. This step was essential because the Passio GO activity data recorded the nearest stop’s name whenever the automated shuttle stopped, making it necessary to distinguish between official and intermediate stops. The computed distance was used to identify whether a stop was an intermediate or official stop.

- Official Stops: If the estimated distance between the stops was equal to the field-recorded distance between two official stops, the stop was treated as an official stop.
- Intermediate Stops: If the distance between stops was less than the field-measured distance, the stop was treated as an intermediate stop. In the case of multiple intermediate stops, the cumulative traveled distance was compared with the field-measured distance between the official stops. If the cumulative traveled distance was less than the field-measured distance, the stops were identified as intermediate stops.

### 5.4. Construction of Shuttle Trajectories

Once the distance between stops, travel time between stops, start time, and exit time were estimated, the automated shuttle trajectory was constructed by assuming an origin point. Seven official stations were identified along the pilot route: GV1, GV8, GV4, SB, SU, SD, and LRT. Due to its central location, the “SB” was chosen as the origin for constructing the trajectories. After processing the trip time and distance, the stop-based data were used to construct the trajectories for the automated shuttle, utilizing the timestamps and the cumulative distance traveled between stops.

### 5.5. Pattern Recognition for Shuttle Routing

Following the construction of automated shuttle trajectories based on stop data, a pattern recognition algorithm was employed to identify potential routing patterns utilized by the automated shuttle. This algorithm was used to analyze the sequence of stops within a trajectory and categorizes trips into distinct routing patterns by comparing these stop sequences. This approach identifies recurring routes that the shuttles take, which can be valuable for understanding operational strategies and potential optimization opportunities.

### 5.6. Integrating Disengagement, Weather, and Roadway Data with Constructed Trajectories

Disengagement data were initially processed to ensure that only disengagements along the official pilot route were accounted for. To do this, a 50-foot (15.24 m) linear buffer was created along the pilot route, and the location of the disengagements was overlaid on this layer to eliminate any disengagements recorded outside this buffer. The processed disengagements were then integrated with the constructed trajectories by matching the timestamps in both the datasets. Given that the GPS data were stop-based, the entry time and exit time for each stop and segment were used to match the disengagement data with the GPS data, ensuring accurate alignment.

Similarly, a matching process was implemented to integrate the weather data with the constructed trajectory. The GPS data included timestamps, while the weather data from Visual Crossing had detailed hourly timestamps. By aligning these timestamps, each GPS data point was matched with the corresponding weather condition at that specific time. This allowed for a comprehensive analysis of how weather influenced the shuttle’s operational performance.

Roadway characteristics data, including the number of crosswalks, intersections, and other roadway features, were integrated with the constructed trajectories based on segment names. This integration enabled the assessment of how different roadway characteristics influenced the shuttle’s operational metrics.

## 6. Methodology

A multi-level mixed-effects model was developed to assess the effects of various factors on the speed of the automated shuttle. The multi-level mixed-effects model was chosen for its ability to handle the nested structure of the data and provide robust estimates by accounting for both fixed and random effects. Unlike standard linear regression models, which assume independence among observations, the mixed-effects model incorporates variability at multiple levels, which is critical for understanding the effect of disengagements in this study. For example, time-of-the-day (TOD) may have a higher effect on the automated shuttle speed in segments with frequent pedestrian activity during peak hours, while road geometry factors such as intersections and crosswalks exert greater influence on the automated shuttle speed in segments with complex layouts.

The dependent variable Y (speed) was assumed to follow a normal distribution and is modeled as a function of several covariates X_i_ (number of intermediate stops; TOD; day-of-the-week (DOW); disengagements; roadway characteristics; and weather). To ensure positive speed, a log-link function was assumed. The general form of the fixed effects model is expressed as follows:

log(E(Y)) = β_0_ + β_1_ X_1_ + β_2_ X_2_ …… + β_n_ X_n_ + ϵ_i_ (1)

where E(Y) is the expected value of the dependent variable; β_0_ is the intercept; X_1_, …, X_n_ are the covariates; and β_1_, …, β_n_ are the coefficients associated with each covariate.

A multi-level mixed-effects Gaussian regression model with a log-link function accounts for variability at different levels. This model includes both fixed effects and random effects. The random effects account for variability specific to individual segments of the route. The general form of the multi-level mixed-effects model is expressed as follows:

log(E(Y_ij_)) = β_0_ + β_1_ X_1ij_ + β_2_ X_2ij_ …… + β_n_ X_nij_ + u_oi_+u_1i_ × Z_1ij_ (2)

where E(Y_ij_) is the expected value of the dependent variable for observation j within the group i, β_0_ is the intercept, β_1_, …, β_n_ are the coefficients associated with each covariate, X_1ij_, … X_nij_ are the covariates for observation j within group i, u_oi_ is the random intercept for group i, and u_1i_ is the random coefficient for covariate Z_1ij_ within group i.

In this model, u_oi_ and u_1i_ have random effects that capture variability across different groups. These random effects are assumed to follow a normal distribution, with mean zero and variances σ^2^_uo_ and σ^2^_u1_, respectively.

u_oi_ ~ N(0, σ^2^_uo_) (3)

u_1i_ ~ N(0, σ^2^_u1_) (4)

The multi-level mixed-effects model with a Gaussian distribution and log-link function provides a more flexible framework that can capture the hierarchical structure of the data, allowing for the effects of covariates to vary across different levels or groups. This is particularly useful in accounting for the random variation in the effects of certain covariates, such as the impact of disengagements, which may differ across segments.

To determine whether the multi-level mixed-effects model offers a significant improvement over the fixed effect model, a Likelihood Ratio (LR) test was conducted. The LR test compares the goodness-of-fit of the two models by evaluating whether the inclusion of random effects significantly improves the model fit. A significant LR test result indicates that the multi-level mixed-effects model better fits the data than the fixed effect model, justifying the incorporation of random effects at the segment level.

## 7. Results

### 7.1. Routing Patterns and Constructed Trajectory

The trajectory construction framework was applied to the stop-based GPS data of the automated shuttle to generate vehicle trajectories. Three different routing patterns for the automated shuttle were observed based on the developed trajectories and the pattern recognition algorithm. In pattern 1, the automated shuttle traveled from the SB to LRT station, then to Greek Village (GV1, GV4, and GV8), and returned to the SB. Pattern 2 involves a shorter route, where the shuttle traveled from the SB to LRT station, and then directly back to the SB. Pattern 3 depicts a route where the shuttle traveled from the SB to LRT station. Routing patterns 1 and 2 for the automated shuttle appear flexible and adaptable, potentially changing based on passenger requests and demand. In contrast, routing pattern 3 for the automated shuttle is consistently observed at the end of the shift or day, likely involving a trip to the parking lot for charging before the next shift.

**Figure 2:**
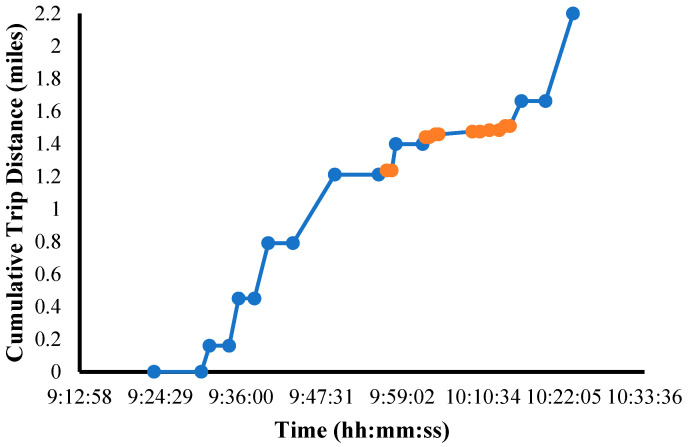
Constructed trajectory of automated shuttle from stop-based GPS data. Note: blue points: official stops; orange points: intermediate stops.

For routing pattern 1, the constructed trajectory for the automated shuttle on 5 December 2023 in the morning is illustrated in Figure 2. The constructed vehicle trajectory was leveraged to estimate performance metrics such as speed/travel time between official stops, number of intermediate stops between two official stops, stop time at each intermediate stop, and stop time at the official stop.

### 7.2. Spatio-Temporal Variation in the Speeds of the Automated Shuttle

**Figure 3:**
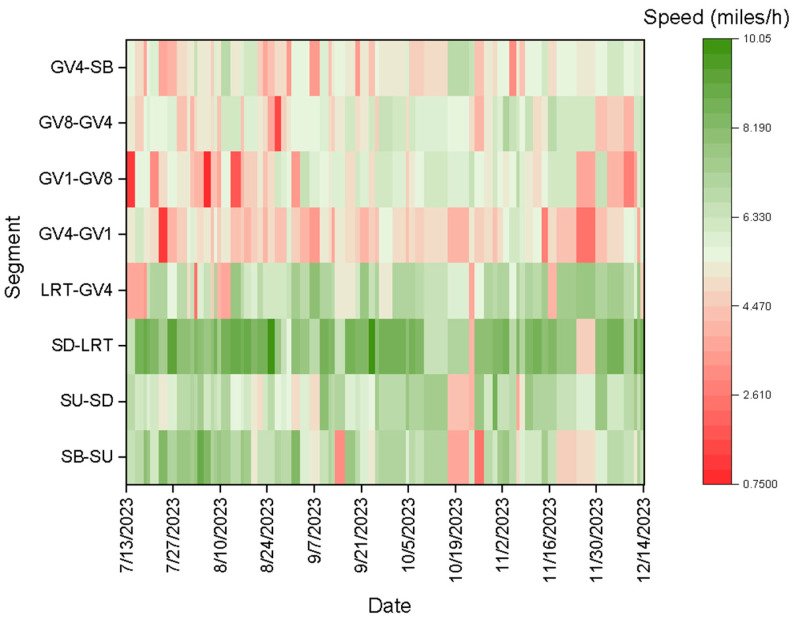
Spatio-temporal variation in automated shuttle speed.

Figure 3 illustrates the spatio-temporal variation in speed for the automated shuttle.

Segments such as SB to SU, SU to SD, and GV4 to SB exhibit high volumes and heterogeneous traffic conditions, characterized by significant pedestrian volumes and vehicle interactions. In these segments, the automated shuttle’s speed is significantly lower compared to in other segments. High pedestrian activity, especially in residential areas like Greek Village (GV1, GV4, and GV8), further contributes to the automated shuttle maintaining lower speeds to prevent crashes and respond effectively to sudden pedestrian movements. This speed reduction is primarily due to the need for careful navigation through crowded and dynamic environments to ensure safety and smooth operation.

The automated shuttle traveled at relatively higher speeds in segments without signalized intersections or traffic signals (SD to LRT) than in those with signalized intersections. This difference is attributed to the absence of frequent stops and signal-induced delays in non-signalized segments, allowing for smoother and more continuous travel. Conversely, segments with signalized intersections required the shuttle to stop or slow down at traffic signals, reducing the overall speed.

Overall, the effect of traffic flow, heterogeneous conditions, and the presence or absence of traffic signals on the speed of the automated shuttle is significant. Lower speeds were observed in areas with high traffic volumes and pedestrian activity to prioritize safety and maneuverability, while higher speeds were observed in less congested and non-signalized areas. This adaptive behavior underscores the automated shuttle’s approach to balancing efficiency with safety in varying traffic environments.

### 7.3. Spatial–Temporal Variation in Disengagements

Disengagements were categorized into four types: environmental factors, technical and signal issues, safety and interaction with others, and navigation and path deviation based on possible causes. Table 1 provides an in-depth analysis of spatio-temporal variation in disengagements by type. The data revealed critical insights into the operational challenges and trends affecting the automated shuttle’s performance.

Disengagements due to environmental factors were prevalent in specific segments and showed a strong temporal pattern. For instance, the segment from GV1 to GV8 recorded 22 disengagements in August, primarily attributed to environmental conditions such as overgrown vegetation obstructing the automated shuttle’s sensors. Vegetation emerged as a key cause of environmental disengagements, underscoring the effect of dynamic seasonal changes on the automated shuttle’s operations. In response, the campus took proactive measures in August to trim overgrown branches along the pilot route, resulting in a significant reduction in vegetation-related disengagements in the subsequent months. These findings highlight the importance of ongoing route maintenance and adaptive strategies to mitigate environmental challenges.

Technical and signal-related disengagements were most frequent across all segments, particularly between SU and SD and GV4 and SB, where signalized intersections posed recurring challenges. These issues were often caused by interruptions in V2I communication, leading to the automated shuttle’s inability to respond to real-time traffic signals effectively. The data underscores the critical need for more reliable communication systems and infrastructure upgrades to ensure seamless interactions between the automated shuttle and its environment. Future technological improvements, such as advanced signal prioritization systems and redundant communication channels, could help address these recurring challenges.

Disengagements triggered by safety concerns and interactions with other road users were most prominent in high-traffic segments like SU–SD and GV4–SB. Unpredictable behaviors from pedestrians, cyclists, and other vehicles often required the safety operator to take control, particularly in areas with high pedestrian activity. These findings emphasize the need for autonomous systems to adopt advanced predictive algorithms that can anticipate and respond to dynamic road-user behaviors in real time, reducing the reliance on manual interventions.

Navigation and path deviation disengagements were less frequent, but significant in segments like GV4–SB and SU–SD. These events were often caused by unclear lane markings or GPS inaccuracies that hindered the automated shuttle’s ability to maintain its intended path. Improving localization accuracy through advanced GPS, redundant positioning technologies, and better roadway markings could mitigate these occurrences, enhancing the overall reliability of autonomous operations.

The segment between SU and SD consistently experienced the highest number of disengagements, peaking in December with 28 incidents, largely due to technical and signal issues at intersections and frequent safety-related interactions with road users. Similarly, the segment from GV4 to SB exhibited high disengagement rates in the summer months, highlighting the combined influence of technical challenges and safety concerns in these critical areas.

### 7.4. Impact of Various Factors on Running Speed

A comparative analysis was conducted to evaluate the performance of the multi-level mixed-effects Gaussian regression model against two alternative models: a standard linear regression model and a generalized linear model (GLM). The results are summarized in Table 2.

The comparative analysis demonstrated that the multi-level mixed-effects Gaussian regression model had the best fit for the data, and the results are summarized in Table 3. The R^2^ is 0.84. The results from this model offer a comprehensive understanding of how various factors influence the speed of the automated shuttle.

#### 7.4.1. Number of Intermediate Stops

The number of intermediate stops significantly influences the operational speed of the automated shuttle. The analysis indicates that each additional intermediate stop reduces the automated shuttle’s speed, with the magnitude of this reduction increasing as the number of stops increases. For instance, having one intermediate stop decreases the expected log of the automated shuttle’s speed by 0.398 units compared to having no intermediate stops. This reduction is even more pronounced with two and three intermediate stops, which decrease the expected log of the speed by 0.738 and 0.968 units, respectively. These findings highlight the practical implications of intermediate stops, as each stop necessitates deceleration, stopping, and subsequent acceleration, thereby reducing the average speed of the shuttle.

#### 7.4.2. Roadway Factors

The operational speed of the automated shuttle is influenced by several roadway factors, including the proportion of divided roads, the number of crosswalks, and the number of intersections. The positive coefficient of 1.025 indicates that an increase in the proportion of divided roads significantly enhances the automated shuttle’s speed. For each unit increase in the proportion of divided roads, the expected log of the speed increases by 1.025 units. Divided roads typically offer a safer and less obstructed environment, allowing for smoother and more continuous travel. The physical barrier between opposing lanes reduces interruptions and potential collisions, enabling the automated shuttle to maintain a higher average speed.

The negative coefficients for the number of crosswalks indicate that more crosswalks reduce the automated shuttle speed. Specifically, one crosswalk decreases the expected log of the speed by 0.339 units, and two crosswalks further decrease it by 0.493 units. Crosswalks require the shuttle to reduce speed to ensure pedestrian safety, leading to lower operational speeds.

The number of intersections also significantly influences the automated shuttle speed. One intersection reduces the expected log of the speed by 0.240 units, three intersections reduce the expected log of the speed by 0.259 units, and four intersections reduce the expected log of the speed by 0.494 units. Intersections typically involve stops or slowdowns due to traffic control devices and interactions with other vehicles, thereby reducing the automated shuttle’s average speed.

#### 7.4.3. Weather

The coefficient for high visibility is 0.009, indicating a very slight positive effect on the automated shuttle speed. While the positive coefficient suggests a relatively higher speed in high visibility conditions, the effect is minimal and not statistically significant. This could imply that the automated shuttle’s navigation system is robust enough to maintain consistent performance under different visibility conditions (as observed in this study) or that the visibility variations during the study period were not severe enough to affect speed substantially. Since the automated shuttle did not operate under inclement weather conditions, such as heavy rainfall, the effect of weather-related parameters, like visibility and precipitation, was insignificant. This limitation highlights the need for further research under more varied and extreme weather conditions to understand their impact on shuttle performance.

#### 7.4.4. Temporal Factors

The analysis by TOD and DOW revealed that these variables have minimal impact on the automated shuttle’s operational speed. The slight variations in speed across different times of the day and days of the week suggest that the automated shuttle maintains a consistent performance.

#### 7.4.5. Disengagements

**Figure 4:**
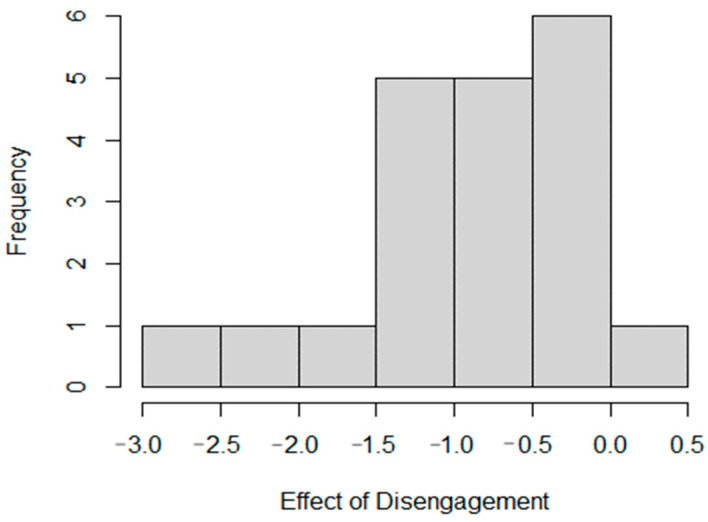
Impact of disengagement.

The negative coefficient of −0.803 indicates that disengagements significantly reduce the automated shuttle’s speed. Specifically, when a disengagement event occurs, the expected log of the speed decreases by 0.803 units. This substantial reduction highlights the disruptive impact of disengagements on the automated shuttle performance. The perfect negative correlation (−1.000) between the intercept and disengagement effects suggests that segments with higher baseline speeds experience a more pronounced reduction in speed when disengagements occur. The variance of the random effect for disengagements across segments is 0.981, with a standard deviation of 0.990, indicating substantial variability in how disengagements impact speed in different segments. Figure 4 illustrates the effect of disengagements across different segments.

The histogram shows the distribution of coefficients for the impact of disengagements on the automated shuttle speed across various segments. Most coefficients are negative, indicating that disengagements generally reduce shuttle speed. The peak frequency of coefficients falls between −1.0 and −0.5, suggesting that for most segments, disengagements significantly reduce the expected log of speed by approximately 0.5 to 1.0 units. The range of coefficients, from about −2.5 to 0.5, indicates variability in the effect, with some segments experiencing more severe speed reductions than others. The distribution is skewed towards the negative, confirming that disengagements predominantly disrupt shuttle operations by necessitating slowdowns or stops for safe manual control transitions. This variability suggests that certain segments, likely due to different traffic conditions or road geometries, are more challenging for autonomous navigation.

In practical terms, disengagements often require the automated shuttle to switch control from the automated system to a human operator. This transition typically involves slowing down or stopping the vehicle to ensure safety, reducing the average speed. The necessity for human intervention during disengagements underscores the challenges faced by autonomous shuttles in maintaining consistent performance in dynamic environments.

## 8. Conclusions

This study provides a comprehensive analysis of the effect of disengagements on the operational performance of automated shuttles, explicitly focusing on speed. The study addresses a critical gap in understanding how disengagement events affect the efficiency of automated shuttles, particularly in complex environments such as university campuses. By employing a multi-level mixed-effects Gaussian regression model, the study effectively quantifies the effect of disengagements while controlling covariates such as roadway geometry, weather conditions, TOD, DOW, and the number of intermediate stops. The following are some of the important conclusions drawn from the study:

- Signalized intersections, high pedestrian activity, and environmental factors like vegetation significantly affected the automated shuttle performance in terms of the frequency of disengagements. Addressing technical issues, enhancing safety protocols, and managing environmental factors are crucial for optimizing the automated shuttle’s performance and reliability, reducing manual interventions, and improving overall service reliability;
- Roadway geometry factors such as the number of crosswalks, the proportion of divided roads, and the number of intersections significantly influence the speed of the automated shuttle. An increase in the number of crosswalks and intersections decreases the speed of the automated shuttle;
- Disengagements significantly reduce the automated shuttle speed, underscoring the disruptive nature of disengagements on automated shuttle performance and emphasizing the need for improvements in autonomous systems to minimize these occurrences. The effects of disengagements varied with location, highlighting the influence of segment-specific characteristics, such as traffic conditions and road geometries, on automated shuttle performance.

While the findings from this study serve as valuable insights, the study has several limitations that warrant future research. The analysis relied on stop-based GPS data, which limited the ability to evaluate the immediate effect of disengagements on shuttle behavior and the reaction time of the safety operator. Continuous GPS tracking and higher-resolution data could provide a more granular understanding of disengagement dynamics. Additionally, the study focused on a specific university campus setting, which, while valuable, may not fully represent operational conditions in larger urban areas or more diverse environments. Extreme weather conditions, such as heavy rain or fog, were not encountered during the study period, leaving gaps in understanding the automated shuttle’s performance under such conditions. Finally, the study analyzed the effect of disengagements on speed within the affected segment, but did not explore the potential propagation of these effects to subsequent segments, nor account for the micro-level variability in human driver behavior during disengagements.

In terms of real-world implementation, significant challenges remain. Scaling the findings to more dynamic and unpredictable environments, such as urban centers with heavy traffic and complex pedestrian interactions, requires addressing the current limitations in autonomous system technologies. Robust sensor capabilities, improved V2I communication, and the ability to handle unexpected situations in real time are essential for broader deployment and a reduction in disengagements. Validation of these results in diverse settings, including areas with varying traffic regulations, infrastructure, and user demographics, is crucial for ensuring the reliability of automated shuttles in public transportation networks. Strategies such as tailored driver training, advanced human–machine interfaces, and predictive models to anticipate human responses could help mitigate the variability of human driver intentions during disengagements.

Future research should prioritize these challenges by exploring the role of predictive models and machine learning techniques in reducing disengagement occurrences and enhancing shuttle decision-making. Investigating the effects of disengagements in diverse operational and environmental conditions using continuous GPS-based and camera-based natural driving datasets, modeling behaviors, assessing interactions with other users (including vehicles), and developing real-time mitigation strategies will be critical. Additionally, pilot programs in varied real-world environments can offer practical insights into overcoming these barriers.

By addressing these limitations and implementation challenges, this study provides a foundation for advancing the practical application and scalability of automated shuttles, contributing to the development of safer, more reliable, and efficient autonomous transportation systems.

## Figures and Tables

**Table 1:** Variation in disengagement type by space and time.

Segment	Category	July	Aug.	Sep.	Oct.	Nov.	Dec.
SB–SU	Environmental factors	5	-	-	-	-	-
	Safety and interaction with others	5	1	-	2	3	-
	Technical and signal issues	1	-	1	-	-	-
	Segment total	11	1	1	2	3	-
SU–SD	Environmental factors	-	-	-	1	1	1
	Navigation and path deviation	-	1	-	-	-	-
	Safety and interaction with others	3	-	3	4	2	3
	Technical and signal issues	13	8	9	6	3	24
	Segment total	16	9	12	11	6	28
SD–LRT	Environmental factors	-	3	-	-	-	1
	Safety and interaction with others	1	1	-	-	-	1
	Technical and signal issues	-	-	-	-	-	3
	Segment total	1	4	-	-	-	5
LRT–GV4	Environmental factors	1	-	-	-	1	-
	Safety and interaction with others	-	-	-	1	-	-
	Technical and signal issues	1	3	7	-	7	2
	Segment total	2	3	7	1	8	2
GV1–GV8	Environmental factors	1	11	-	1	-	-
	Safety and interaction with others	1	11	-	2	1	-
	Technical and signal issues	1	-	1	-	-	-
	Segment total	3	22	1	3	1	-
GV8–GV4	Environmental factors	-	-	-	-	1	-
	Safety and interaction with others	-	-	-	1	-	1
	Segment total	-	-	-	1	1	1
GV4 SB	Environmental factors	5	2	-	2	1	-
	Navigation and path deviation	5	-	3	1	-	1
	Safety and interaction with others	2	3	3	7	4	3
	Technical and signal issues	2	15	12	-	4	3
	Segment total	14	20	18	10	9	7
Total		47	59	39	28	28	43

Note: SB—Science Building; SU—Student Union West; SD—Student Union Deck; LRT—Light Rail Transit Main Station; GV4—Greek Village 4; GV1—Greek Village 1; and GV8—Greek Village 8.

**Table 2:** Model comparison.

Model	Likelihood	AIC	BIC
Gamma log	−4599.08	9240.15	9361.16
Gaussian identity	−4121.72	8291.44	8375.89
Gaussian log	−4055.6	8155.2	8282

**Table 3:** Model summary.

Fixed Effects				
Predictor	Estimate	St. Error	*t*-Statistic	*p*-Value
(Intercept)	1.647	0.039	42.007	<2 × 10^−16^
Proportion of Divided Road	1.025	0.157	6.522	0.000
Number of Intermediate Stops_1	−0.398	0.029	−13.832	<2 × 10^−16^
Number of Intermediate Stops_2	−0.738	0.129	−5.703	0.000
Number of Intermediate Stops_3	−0.968	0.143	−6.786	0.000
Number of Crosswalks_1	−0.339	0.147	−3.358	0.001
Number of Crosswalks_2	−0.493	0.034	9.939	<2 × 10^−16^
Number of Intersections_1	−0.240	0.048	−4.963	0.000
Number of Intersections_3	−0.259	0.029	−8.975	<2 × 10^−16^
Number of Intersections_4	−0.494	0.099	−4.974	0.000
Disengagement	−0.803	0.246	−3.259	0.001
Afternoon Period	−0.008	0.009	−0.875	0.382
Evening Period	0.010	0.020	0.516	0.606
Visibility_High	0.009	0.015	0.586	0.558
Tuesday	0.015	0.014	1.067	0.286
Wednesday	0.036	0.013	2.741	0.006
Thursday	−0.014	0.014	−0.976	0.329
Friday	0.023	0.014	1.671	0.095
Random Effects				
Group	Name	Variance	Std. Dev.	Corr
Location/Segment	(Intercept)	0.018	0.133	
Location/Segment	Disengagement	0.981	0.990	−1.000
Goodness-of-fit				
AIC	8155.2			
BIC	8282			
Log Likelihood	−4055.6			
Deviance	8111.2			
R^2^	0.84			
Likelihood Ratio Test				
Chi-square	82.782			
*p*-value	<2.2 × 10^−16^			

## Data Availability

Data are restricted but could be shared if the request is reasonable.
